# Robust Mesh Denoising via Triple Sparsity

**DOI:** 10.3390/s19051001

**Published:** 2019-02-26

**Authors:** Saishang Zhong, Zhong Xie, Jinqin Liu, Zheng Liu

**Affiliations:** 1 Faculty of Information Engineering, China University of Geosciences, Wuhan 430074, China; saishang@cug.edu.cn (S.Z.); xiezhong@cug.edu.cn (Z.X.); ljqcug@163.com (J.L.); 2 National Engineering Research Center of Geographic Information System, China University of Geosciences, Wuhan 430074, China

**Keywords:** mesh denoising, triple sparsity, Kinect, variable-splitting, augmented Lagrange method

## Abstract

Mesh denoising is to recover high quality meshes from noisy inputs scanned from the real world. It is a crucial step in geometry processing, computer vision, computer-aided design, etc. Yet, state-of-the-art denoising methods still fall short of handling meshes containing both sharp features and fine details. Besides, some of the methods usually introduce undesired staircase effects in smoothly curved regions. These issues become more severe when a mesh is corrupted by various kinds of noise, including Gaussian, impulsive, and mixed Gaussian–impulsive noise. In this paper, we present a novel optimization method for robustly denoising the mesh. The proposed method is based on a triple sparsity prior: a double sparse prior on first order and second order variations of the face normal field and a sparse prior on the residual face normal field. Numerically, we develop an efficient algorithm based on variable-splitting and augmented Lagrange method to solve the problem. The proposed method can not only effectively recover various features (including sharp features, fine details, smoothly curved regions, etc), but also be robust against different kinds of noise. We testify effectiveness of the proposed method on synthetic meshes and a broad variety of scanned data produced by the laser scanner, Kinect v1, Kinect v2, and Kinect-fusion. Intensive numerical experiments show that our method outperforms all of the compared select-of-the-art methods qualitatively and quantitatively.

## 1. Introduction

Recently, with the development of consumer-grade scanner devices (e.g., Microsoft Kinect, Xtion Pro, Google Project Tango, and Intel RealSense), triangulated meshes can be easily acquired from the real world. The scanned meshes can be further used in a variety of application domains, such as geometry processing, computer vision, virtual reality, cultural heritage preservation, and terrain modeling. However, these scanned meshes are inevitably contaminated by different kinds of noise, introduced by the scanning process and the reconstruction algorithm. The noise can not only degrade the quality of meshes, but also cause errors in downstream geometry applications [1]. Thus, the task of removing noise from scanned meshes becomes increasingly important. The main challenge is to remove noise while preserving both sharp features (including edges and corners) and fine details as well as preventing introducing undesired staircase effects in smooth regions. This problem becomes more difficult when meshes are polluted by different kinds of noise including Gaussian, impulsive, and mixed noise.

Mesh denoising is a fundamental problem in geometry processing, which has been studied for years. Early, filtering methods are wildly applied in mesh denoising. The filtering methods can be divided into two categories: isotropic and anisotropic methods. The isotropic methods [2,3] are classical for their simplicity. Although these methods can remove noise, they often cause significant shape distortion. The reason is that these methods do not consider geometric features during the denoising. Later on, for preserving geometric features, many anisotropic methods were proposed [4,5,6,7,8,9]. When the level of noise is low, the anisotropic methods work well. However, when the noise level increases, these methods tend to blur sharp features. Recently, bilateral filtering methods have been studied in mesh denoising [10,11]. Since these methods also belong to anisotropic methods, they still blur sharp features. In order to preserve sharp features, some works [12,13,14] applied the bilateral filtering in the face normal field. Unfortunately, when the noise level is high, the bilateral normal filtering proposed by Zheng et al. [12] still cannot recover sharp features well. Zhang et al. [13] proposed a normal filtering method based on a well-designed guided normal field. Although their method can preserve sharp features, it lacks robustness to the mesh topology. The robust normal filtering method [14] can also preserve sharp features, but it usually blurs fine details.

Recently, variational methods based on sparsity have been proved successful in image restoration [15,16,17] for the edge-preserving property of them. These methods are inspired by the emerging theories of sparse signal reconstruction and compressive sampling [18,19]. Inspired by these, sparse optimization methods are introduced in mesh denoising [20,21,22,23,24]. He and Schaefer [20] extended $\ell_{0}$ minimization from images to surfaces, which induces sparsity on an edge-based operator. However, the $\ell_{0}$ minimization is NP-hard. The works [21,22] extended total variation (TV) minimization for preserving sharp features of the mesh. To handle irregular sampling meshes corrupted by different kinds of noise, Lu et al. [24] presented an $\ell_{1}$-norm normal filtering method. Although the above sparsity-based methods [20,21,22,23,24] can remove noise while preserving sharp features, they inevitably suffer undesired staircase effects in smoothly curved regions. This problem is even worse for the $\ell_{0}$ minimization [20] for its high sparsity requirement. In order to overcome the staircase effects introduced by these first order methods [20,21,22,23,24], Liu et al. [1] proposed a high order normal filtering method, which can preserve sharp features and simultaneously prevent introducing staircase effects in smooth regions. Unfortunately, when the noise level increases, the high order method [1] sometimes smoothes sharp features.

More recently, researchers proposed some methods based on geometric priors. Assuming the additive noise of the noisy mesh is Gaussian noise, a method based on compressed sensing was proposed to decouple features and the noise [25]. However, if the noise level is high, it is difficult for this method to distinguish features from the noise. With the assumption of geometric features are not seriously corrupted by the noise, Lu et al. [23] first detected geometric features from a pre-filtered mesh, and then they reconstructed the denoised result by the detected features. On the contrary, without any assumptions about the underlying surface, a data-driven method has been employed for mesh denoising [26]. The method first learns non-linear regression functions mapping filtered face normal descriptors to face normals of the clean mesh, and then employs the learned functions for computing the filtered face normals. This method can effectively remove noise and preserve geometric features. Yet, it is very dependent on the completeness of the training data set.

As we can see, the above mentioned mesh denoising methods have their own limitations. In summary, except the method [1], filtering methods and sparse optimization methods are either preserve fine details or sharp features well. Moreover, without considering the noise type, these methods are difficult to handle different kinds of noise, which often exist in the real data acquired by consumer-grade scanners. To a certain extent, these problems will degrade the quality of denoising results. To overcome the above limitations, we present a two-stage mesh denoising method. At the first stage, we propose a variational normal filtering model based on a triple sparsity prior. After that, we evolve the mesh to match the filtered face normals at the second stage. Taking a noisy mesh as the input, our method can robustly handle various kinds of noise while preserving geometric features.

Specifically, the contributions of the paper are listed as follows:

- We present a novel normal filtering model with three sparsity terms. The model can recover both sharp features and fine details and simultaneously prevent introducing unnatural effects in smooth regions. Besides, the model is robust against different kinds of noise.
- We develop an efficient algorithm based on variable-splitting and augmented Lagrangian method for solving the problem.
- We demonstrate the performance of our denoising method on synthetic meshes and a variety of scanned data produced by the laser scanner, Kinect v1, Kinect v2, and Kinect-fusion. Our method outperforms compared methods for both synthetic meshes and real scanned data.

The rest of the paper is organized as follows. In Section 2, we first propose a variational normal filtering model based on a triple sparsity prior. Then, an iterative algorithm using augmented Lagrange method and variable-splitting technique is presented to solve the problem. Finally, according to the filtered face normals, the vertex positions are updated by a robust vertex updating scheme. The comparisons about our mesh denoising method and state-of-the-art methods are demonstrated in Section 3. Section 4 concludes the paper.

## 2. Robust Mesh Denoising

Similarly to some previous mesh denoising methods [9,12,13,14,21], our method belongs to two-stage methods, i.e., face normal filtering followed by updating vertices.

### 2.1. Normal Filtering

In this subsection, we first briefly give some necessary notations, and then introduce our normal filtering method. A mesh of arbitrary topology with no degenerate triangles in $R^{3}$ is represented as *M*. The set of vertices, edges, and triangle faces of *M* are denoted as $\left\{v_{i}:i=1,2,\ldots ,V\right\}$, $\left\{e_{i}:i=1,2,\ldots ,E\right\}$, and $\left\{\tau_{i}:i=1,2,\ldots ,T\right\}$, respectively. Here, V, E, and T are the numbers of vertices, edges, and faces of *M*. Furthermore, we denote the 1-disk of vertex $v_{i}$ as $D_{1}\left(v_{i}\right)$, which is the set of triangles containing $v_{i}$.

To filter the face normals of the noisy input, we propose a normal filtering model containing three sparsity terms. It consists of a double sparsity prior on first order and second order variations of the face normal field to recover sharp features, fine details, and smooth regions and a third sparsity prior for handling different kinds of noise. Besides, we also present an iterative algorithm to solve the proposed normal filtering model.

#### 2.1.1. Normal Filtering Model

**Figure 1:**
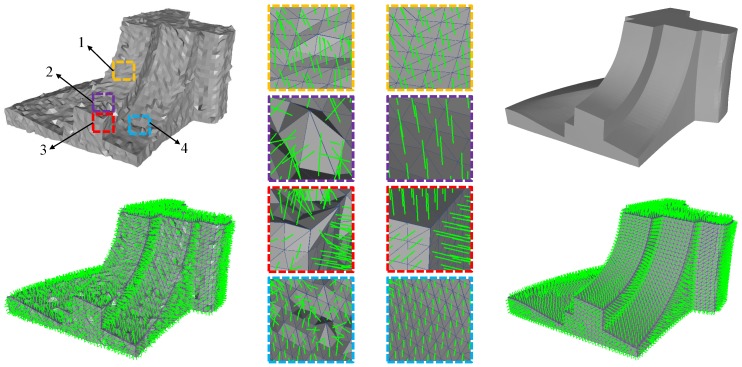
The illustration of effects of the four terms in our normal filtering model. The first column shows the noisy mesh (**top**) and the face normals of it (**bottom**). The second column shows the face normals of four magnified patches of the noisy mesh, while the third column shows the face normals of the corresponding magnified patches of the denoising result. The fourth column shows the denoising result (**top**) and the face normals of it (**bottom**).

Given a noisy mesh $M^{in}$, we represent its face normals as $N^{in}$. To filter the noise of $N^{in}$, we treat the face normals N as a variable and propose the following normal filtering model:

(1) $$\min_{N\in C_{N}}\;\left\{\alpha E_{f_{2}}+\beta E_{f_{1}}+\gamma E_{tv}+\delta E_{aho}\right\},$$

where $C_{N}=\{N\in R^{3\times T}:∥N_{\tau }{∥}_{2}=1,\forall \tau \}$, α,β,γ, and δ are positive parameters used to balance the four terms including one $\ell_{2}$-norm term and three $\ell_{1}$-norm terms. The first two terms are used to control the degree of denoising, while the last two terms are used to regularize the noisy mesh for noise removal and feature preserving. In the following, we will introduce the effects of these four terms with Figure 1.

**$\ell_{2}$-norm fidelity term $E_{f_{2}}$:**

(2) $$E_{f_{2}}=\sum_{\tau }a\left(\tau \right){∥N_{\tau }-N_{\tau }^{in}∥}^{2},$$

where a(τ) is the area of triangle τ. The $\ell_{2}$-norm fidelity term is used to make the solution to harmonise well with the input face normals. It is well known that this least square fidelity term is used for additive Gaussian noise. As we can see in the first pair of magnified views of Figure 1, within the patch corrupted by Gaussian noise, this least square fidelity term can keep the solution of the face normals (see the magnified view on the right) close to the input face normals (see the magnified view on the left).

**$\ell_{1}$-norm fidelity term $E_{f_{1}}$:**

(3) $$E_{f_{1}}=\sum_{\tau }a\left(\tau \right)∥N_{\tau }-N_{\tau }^{in}∥.$$

Similarly to the $\ell_{2}$-norm fidelity term, the $\ell_{1}$-norm fidelity term also encourages the solution to be close to the input face normals. This $\ell_{1}$-norm fidelity term is less well known. It can be used to avoid the influence of outliers for impulsive noise. As we can see in the second pair of magnified views of Figure 1, this $\ell_{1}$-norm fidelity term encourages replacing the outliers with less dependence on their exact value. In other words, this fidelity term make the regularization be robust against outliers for impulsive noise.

**TV regularization term $E_{tv}$:**

$$E_{tv}=\sum_{e}{\mathrm{len}\left(e\right)∥\nabla N|}_{e}∥,$$

where len(e) is the length of edge *e*, and ∇ is a discrete gradient operator defined over triangulated meshes. This first order operator (gradient operator) is defined on each edge of the mesh, and its computation can refer to Ref. [21].

The TV regularization has been proven very successful in image processing for its excellent edge-preserving property [21]. We extend it to mesh denoising for preserving sharp features (including edges and corners) while removing noise. As can be seen in Figure 1, the TV regularization term can remove undesired geometric oscillations at the edges and corners of the mesh (see the third pair of magnified views). Thus, this TV regularization term enables sharp features preserving while removing noise. However, the TV regularization tends to optimize the face normal field to be a piecewise constant field, which introduces undesired staircase effects in smooth regions [1]. These undesired staircase effects will degrade the quality of denoising results.

**Anisotropic high order regularization term $E_{aho}$:**

(4) $$E_{aho}=\sum_{l}{\mathrm{len}\left(l\right)∥D\left(N\right)|}_{l}∥,$$

where len(l) is the length of line *l* connecting the barycenter with one vertex of triangle τ. The anisotropic second order operator D is defined on each line of the mesh, which reads as follows

$${D\left(N\right)|}_{l}=w_{e^{+}}\left(N_{\tau }-N_{\tau^{+}}\right)+w_{e^{-}}\left(N_{\tau }-N_{\tau^{-}}\right),$$

where $e^{+}$ and $e^{-}$ are two edges sharing the common vertex of *l*, $\tau^{+}$ is the triangle sharing $e^{+}$ with τ, and $\tau^{-}$ is the triangle sharing $e^{-}$. For more details about these descriptions, we refer readers to [1]. $w_{e^{+}}$ and $w_{e^{-}}$ are positive weights defined as

(5) $$w_{e}=\exp(-∥N_{e,1}-N_{e,2}{∥}^{2}/2\sigma^{2}),$$

where $N_{e,1}$ and $N_{e,2}$ are the normals of two faces sharing the common edge *e*. We should point out that, we discretize the second order operator D in an anisotropic manner. In contrast, the discretization of the second order operator in Ref. [1] is isotropic. Compared to the discretization in Ref. [1], our discretization has better feature-preserving property.

As mentioned before, the TV regularization term will introduce undesired staircase effects in smooth regions. In order to overcome this problem, we use the anisotropic high order regularization (4) to recover the smooth regions while preventing introducing the staircase effects; see the fourth pair of magnified views of Figure 1 for example. Moreover, the anisotropic high order regularization will not blur sharp features during the smoothing process.

#### 2.1.2. Augmented Lagrangian Method for Solving the Normal Filtering Model

Because of the nondifferentiability and nonlinear constraints of the model (1), it is difficult to directly solve it. Recently, variable-splitting and augmented Lagrangian method (ALM) have achieved great success in $\ell_{1}$ related optimization problems [1,21,22]. Here, we introduce three auxiliary variables and employ ALM to solve the problem. Furthermore, since the weights (5) are estimated from the noisy input, we dynamically update them at each iteration to improve the quality of denoising results.

We first introduce three auxiliary variables X,Y, and Z, and then reformulate the problem (1) as

$$\begin{array}{cc} \min_{N,X,Y,Z} & \left\{\alpha E_{f_{2}}+\beta F\left(X\right)+\gamma R\left(Y\right)+\delta Q\left(Z\right)+\Phi \left(N\right)\right\},\\ & s.t.,\;X=N\;-\;N^{in},\;Y=\nabla N,\;Z=D\left(N\right), \end{array}$$

where $F\left(X\right)=\sum_{\tau }a\left(\tau \right)∥X_{\tau }∥,R\left(Y\right)=\sum_{e}\mathrm{len}\left(e\right)∥Y_{e}∥$, $Q\left(Z\right)=\sum_{l}\mathrm{len}\left(l\right)∥Z_{l}∥$, and

$$\Phi \left(N\right)=\left\{\begin{array}{cc} 0,\; & N\in C_{N}\\ +\infty ,\; & N\notin C_{N.} \end{array}\right.$$

To solve the above constrained optimization problem, we define the following augmented Lagrangian function

(6) $$\begin{array}{cc} L(N,X,Y,Z;\lambda_{x},\lambda_{y},\lambda_{z})= & \alpha E_{f_{2}}+\beta F\left(X\right)+\gamma R\left(Y\right)+\delta Q\left(Z\right)+\Phi \left(N\right)+\sum_{\tau }a\left(\tau \right)\lambda_{x,\tau }\;\cdot \;\left(X_{\tau }\;-\;\left(N_{\tau }\;-\;N_{\tau }^{in}\right)\right)\\ & +\frac{r_{x}}{2}\sum_{\tau }a\left(\tau \right){∥X_{\tau }\;-\;\left(N_{\tau }\;-\;N_{\tau }^{in}\right)∥}^{2}+\sum_{e}\mathrm{len}\left(e\right)\lambda_{y,e}\;\cdot \;\left(Y_{e}\;-\;\nabla N{|}_{e}\right)\\ & +\frac{r_{y}}{2}\sum_{e}\mathrm{len}\left(e\right)∥Y_{e}\;-\;\nabla N{{|}_{e}∥}^{2}+\sum_{l}\mathrm{len}\left(l\right)\lambda_{z,l}\;\cdot \;\left(Z_{l}\;-\;D\left(N\right){|}_{l}\right)\\ & +\frac{r_{z}}{2}\sum_{l}\mathrm{len}\left(l\right)∥Z_{l}\;-\;D\left(N\right){{|}_{l}∥}^{2}, \end{array}$$

where $\lambda_{x}=\left\{\lambda_{x,\tau }\right\}\in R^{3\times T}$, $\lambda_{y}=\left\{\lambda_{y,e}\right\}\in R^{3\times E}$, and $\lambda_{z}=\left\{\lambda_{z,l}\right\}\in R^{3\times L}$ are three Lagrange multipliers, and $r_{x}$, $r_{y}$, and $r_{z}$ are the positive penalty coefficients. Note that L is the number of lines connecting the barycenter and one vertex of triangle τ. We solve the problem (6) by iteratively solving four subproblems: the N-subproblem, X-subproblem, Y-subproblem, and Z-subproblem. In the following, we discuss solutions to these four subproblems.

(1) N-subproblem: the sub-minimization problem of N can be written as

$$\begin{array}{cc} \min_{N} & \alpha E_{f_{2}}+\Phi \left(N\right)+\frac{r_{x}}{2}\sum_{\tau }a\left(\tau \right)∥N_{\tau }\;-\;N_{\tau }^{in}\;-\;\left(X_{\tau }+\frac{\lambda_{x,\tau }}{r_{x}}\right){∥}^{2}+\frac{r_{y}}{2}\sum_{e}\mathrm{len}\left(e\right){∥\nabla N|}_{e}\;-\;\left(Y_{e}+\frac{\lambda_{y,e}}{r_{y}}\right){∥}^{2}\\ & +\frac{r_{z}}{2}\sum_{l}{\mathrm{len}\left(l\right)∥D\left(N\right)|}_{l}\;-\;\left(Z_{l}+\frac{\lambda_{z,l}}{r_{z}}\right){∥}^{2}, \end{array}$$

which is a quadratic optimization with the unit normal constraints Φ(N). We first fix the variables (X,Y, and Z), and then use an approximate strategy to solve this problem. Specifically, we ignore the term Φ(N) and solve the problem

(7) $$\begin{array}{cc} \min_{N} & \alpha E_{f_{2}}+\frac{r_{x}}{2}\sum_{\tau }a\left(\tau \right)∥N_{\tau }-N_{\tau }^{in}-\left(X_{\tau }+\frac{\lambda_{x,\tau }}{r_{x}}\right){∥}^{2}+\frac{r_{y}}{2}\sum_{e}\mathrm{len}\left(e\right){∥\nabla N|}_{e}-\left(Y_{e}+\frac{\lambda_{y,e}}{r_{y}}\right){∥}^{2}\\ & +\frac{r_{z}}{2}\sum_{l}{\mathrm{len}\left(l\right)∥D\left(N\right)|}_{l}-\left(Z_{l}+\frac{\lambda_{z,l}}{r_{z}}\right){∥}^{2}. \end{array}$$

Then, we project the solution of the problem (7) to a unit sphere. Generally, the solution of the quadratic optimization problem (7) can be easily achieved by sparse linear system, which can be solved by using various numerical packages, such as Eigen, Taucs, and Math Kernel Library (MKL).

(2) X-subproblem: the sub-minimization problem of X is given as

(8) $$\min_{X}\;\beta F\left(X\right)+\frac{r_{x}}{2}\sum_{\tau }a\left(\tau \right){∥X_{\tau }-\left(N_{\tau }\;-\;N_{\tau }^{in}\;-\;\frac{\lambda_{x,\tau }}{r_{x}}\right)∥}^{2}.$$

This problem is easy to solve due to the energy function (8) can be spatially decomposed, where the minimization problem w.r.t. each face is performed individually. Thus, for each $X_{\tau }$, we only need to solve the following problem

$$\min_{X_{\tau }}\;\beta ∥X_{\tau }∥+\frac{r_{x}}{2}{∥X_{\tau }-\left(N_{\tau }\;-\;N_{\tau }^{in}\;-\;\frac{\lambda_{x,\tau }}{r_{x}}\right)∥}^{2},$$

which has a closed form solution as

(9) $$X_{\tau }=\mathrm{Shrink}\left(\beta ,\;r_{x},\;N_{\tau }\;-\;N_{\tau }^{in}\;-\;\frac{\lambda_{x,\tau }}{r_{x}}\right),$$

where the Shrink operator is defined as Shrink$\left(u,v,w\right)=\max\left(0,1\;-\;\frac{u}{v∥w∥}\right)w$.

(3) Y-subproblem: the sub-minimization problem of Y is given as

(10) $$\min_{Y}\;\gamma R\left(Y\right)+\frac{r_{y}}{2}\sum_{e}\mathrm{len}\left(e\right){∥Y_{e}-\left(\nabla N{|}_{e}-\frac{\lambda_{y,e}}{r_{y}}\right)∥}^{2}.$$

The sub-problem of Y is separable and can be formulated as edge-by-edge problems. So, for each $Y_{e}$, we have the following simplified problem

$$\min_{Y_{e}}\;\gamma ∥Y_{e}∥+\frac{r_{y}}{2}{∥Y_{e}-\left(\nabla N{|}_{e}-\frac{\lambda_{y,e}}{r_{y}}\right)∥}^{2},$$

which has a closed form solution as

(11) $$Y_{e}=\mathrm{Shrink}\left(\gamma ,\;r_{y},\;\nabla N{|}_{e}-\frac{\lambda_{y,e}}{r_{y}}\right).$$

(4) Z-subproblem: the sub-minimization problem of Z can be formulated as

(12) $$\min_{Z}\;\delta Q\left(Z\right)+\frac{r_{z}}{2}\sum_{l}\mathrm{len}\left(l\right){∥Z_{l}-\left(D\left(N\right){|}_{l}-\frac{\lambda_{z,l}}{r_{z}}\right)∥}^{2}.$$

Since the energy function (12) w.r.t. each line is individually performed, the subproblem (12) can be solved independently. For each $Z_{l}$, we solve the following problem

$$\min_{Z_{l}}\;\delta ∥Z_{l}∥+\frac{r_{z}}{2}{∥Z_{l}-\left(D\left(N\right){|}_{l}-\frac{\lambda_{z,l}}{r_{z}}\right)∥}^{2},$$

which has a closed form solution

(13) $$Z_{l}=\mathrm{Shrink}\left(\delta ,\;r_{z},\;D\left(N\right){|}_{l}-\frac{\lambda_{z,l}}{r_{z}}\right).$$

The entire procedure for solving the problem (6) is outlined in Algorithm 1. The algorithm iteratively solves the above four subproblems and updates the Lagrange multipliers and weights (5). Since the weights (5) estimated from noisy face normals are not accurate, we dynamically update them in each iteration for preserving geometric features better. 

**Algorithm 1:** ALM for Solving Normal Filtering Model (1)

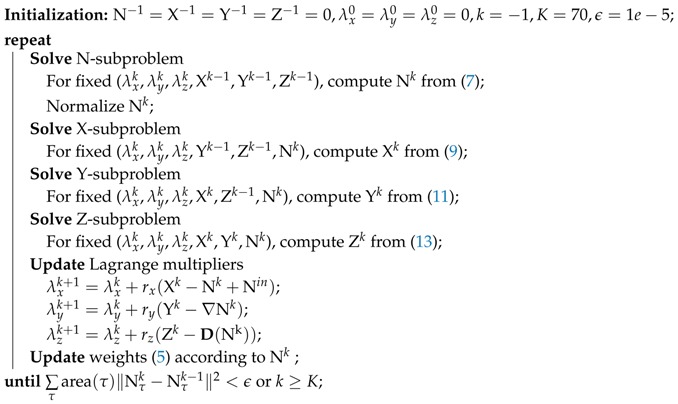



### 2.2. Robust Vertex Updating

After optimizing the face normals by the normal filtering model (1), the vertex positions of the mesh should be updated to match the filtered face normals. To this end, we use a vertex updating scheme presented by Liu et al. [1], which can robustly reconstruct the mesh without foldovers. The method updates the vertex positions by minimizing the following problem

(14) $$\min_{v}\left\{E\left(v\right)=\;\sum_{\tau =(v_{i},v_{j},v_{k})}{\left(N_{\tau }\;-\;\frac{\left(v_{j}\;-\;v_{i}\right)\times \left(v_{k}\;-\;v_{i}\right)}{∥\left(v_{j}\;-\;v_{i}\right)\;\times \;\left(v_{k}\;-\;v_{i}\right)∥}\right)}^{2}\;+\frac{\eta }{2}\|v-v^{in}{\|}^{2}\right\},$$

where $\left(v_{i},v_{j},v_{k}\right)$ are vertices of τ with counterclockwise order, $v^{in}$ is the vertex positions of the noisy mesh and η is a small positive parameter.

We can reformulate the partial derivatives of (14) with respect to $v_{i}$ as follows:

(15) $$\frac{\partial E_{v}}{\partial v_{i}}=\sum_{\tau \in D_{1}\left(v_{i}\right)}\left(N_{\tau }\;-\;\left(N_{\tau }\cdot N_{\tau }\right)N_{\tau }\right)\;\times \;\left(v_{j}\;-\;v_{k}\right)+\eta \left(v_{i}-v_{i}^{in}\right),$$

where $N_{\tau }$ is the updating normal of τ according to the updated *v* (the derivation process of formula (15) can refer to Ref. [1]). With gradient information calculated from (15) and the initial vertex positions, we adopt Broyden-Fletcher-Goldfarb-Shanno (BFGS) algorithm [27] to solve the model (14). In each iteration, BFGS algorithm uses the energy and gradient evaluated at the current and previous iterations.

## 3. Experiment Results and Comparisons

We have implemented our two-stage denoising method on a laptop with a Intel i7 core 2.6 GHZ processor and 8GB RAM. All the tested methods in this paper have been implemented by C++ and run on the same laptop. All of the meshes are rendered in a flat-shading model to show faceting effect. We evaluate our method on various kinds of surfaces including CAD, non-CAD meshes, and real scanned data captured by the laser scanner, Kinect v1, and Kinect v2.

### 3.1. Parameter Setting

As mentioned in Section 2.1, our normal filtering model (1) has four parameters: α,β,γ and δ. The first two parameters are used to control the $\ell_{2}+\ell_{1}$ fidelity terms. The last two are used to balance the first order and second order regularization terms with the $\ell_{2}+\ell_{1}$ fidelity terms. These four parameters need to be tuned by users for producing satisfactory denoising results.

**Figure 2:**
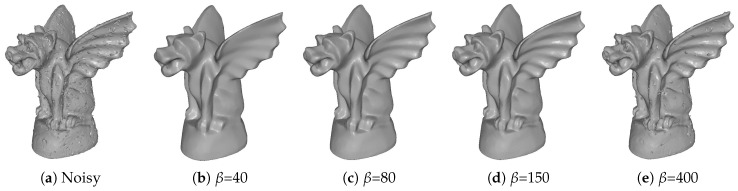
Denoising results for different β with fixed other parameters (α, γ, and δ). From left to right: input noisy mesh (corrupted by Gaussian noise with standard deviation σ=0.1 mean edge length and 15% of impulsive noise with standard deviation σ=0.6 mean edge length) and results with different β.

α and β are introduced to prevent the solution deviating far from the input. α is tuned to handle additive Gaussian noise, and β is tuned to deal with additive impulsive and mixed noise. Due to the influence of these two parameters are similar (both two are used to control the degree of denoising results), we just remark β for an example. Figure 2 shows the results of different β with fixed other parameters. We can see that the details of the mesh gradually appear when β increases. However, if β is too large, the impulsive noise cannot be removed; see Figure 2e.

**Figure 3:**
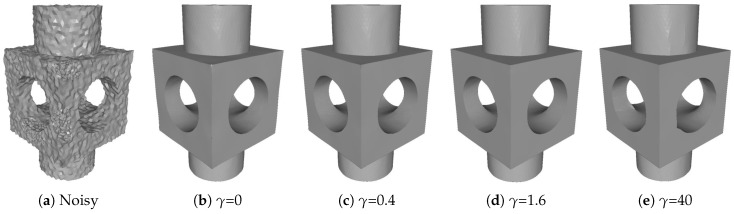
Denoising results for different γ with fixed other parameters (α,β, and δ). From left to right: input noisy mesh (corrupted by Gaussian noise with standard deviation σ=0.2 mean edge length) and results with different γ.

γ controls first order sparsity introduced by the TV regularization term of the model (1). Figure 3 illustrates results of different γ with fixed other parameters. We can see that, if α is zero or too small, some sharp corners are blurred (see Figure 3b). On the contrary, too large α will sharpen some smooth curves and flatten some smoothly curved regions; see Figure 3e. We should point out that, for each input noisy mesh, there exist a range of γ for our method to give visually well denoising results; see Figure 3c,d.

**Figure 4:**
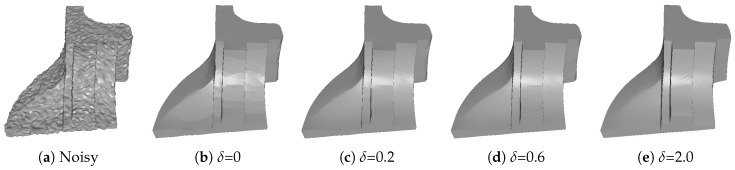
Denoising results for different δ with fixed other parameters (α,β, and γ). From left to right: input noisy mesh (corrupted by Gaussian noise with standard deviation σ=0.2 mean edge length) and results with different δ.

δ influences the smoothness of denoising results. Figure 4 shows the results of different δ with fixed other parameters. As we can see in Figure 4b, if δ is zero or too small, the result suffers undesired staircase effects. In contrast, if δ is too large, the result will be oversmoothed; see the blurred shallow edge in Figure 4e. Again, for each noisy input, there exist a range of δ for our method producing visually well results; see Figure 4c,d.

### 3.2. Qualitative Comparisons

In this subsection, we compare our mesh denoising method (abbreviated as TS) with several state-of-the-art methods including bilateral normal filtering [12], $\ell_{0}$ minimization [20], TV normal filtering [21], robust and high fidelity mesh denoising [14], and high order normal filtering [1], which are abbreviated as localBF/globalBF, $\ell_{0}$, TV, RHM, and HO, respectively. For all of the tested methods, we carefully tune their parameters for producing visually best results.

**Figure 5:**
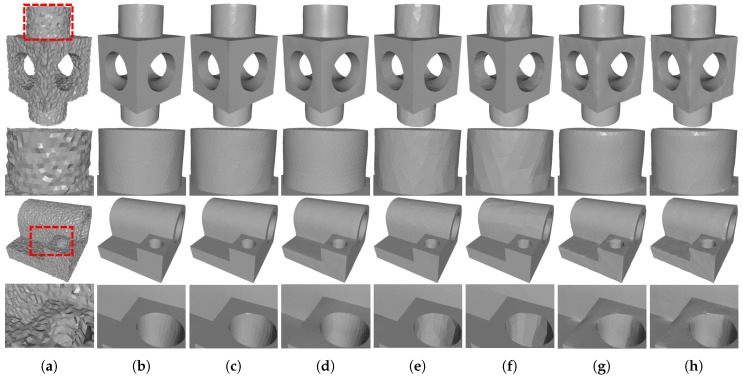
Denoising results of Block and Joint (corrupted by Gaussian noise, standard deviation = 0.2 mean edge length). From left to right: (**a**) noisy meshes, denoising results produced by (**b**) our method TS; (**c**) HO [1]; (**d**) RHM [14]; (**e**) TV [21]; (**f**) $\ell_{0}$ [20]; (**g**) localBF [12]; and (**h**) globalBF [12], respectively. The second and fourth rows are zoomed-in views.

In Figure 5, we compare the denoising results for CAD meshes containing both sharp features and smooth regions. As we can see, all of the tested methods can effectively remove the noise. However, both localBF and globalBF blur sharp features; see Figure 5g,h. This is because that bilateral filters cannot distinguish sharp features from the noise when meshes are corrupted by the high level of noise. On the contrary, the two sparse optimization methods ($\ell_{0}$ and TV) can recover sharp features well. Yet, these two methods suffer staircase effects in smooth regions, see Figure 5e,f. Since $\ell_{0}$ has the highest sparsity requirement, it sometimes generates false edges in smooth regions. Besides, by using the robust error norm, RHM also can preserve sharp features well (see Figure 5d). Although our method TS and HO belong to sparse optimization methods, both of them can not only preserve sharp features but also recover smooth regions; see Figure 5b,c. This is because these two methods use the second order variations of the surface. Furthermore, due to the first order variations are also employed in our method, it can preserve sharp features better than HO. As a result, visual comparisons in Figure 5 show that our method is noticeably better than the other six compared methods in terms of recovering sharp features and smooth regions.

**Figure 6:**
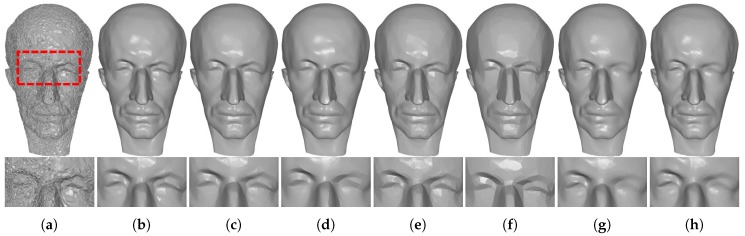
Denoising results of Max-Planck (corrupted by Gaussian noise, standard deviation = 0.2 mean edge length). From left to right: (**a**) noisy mesh, denoising results produced by (**b**) our method TS; (**c**) HO [1]; (**d**) RHM [14]; (**e**) TV [21]; (**f**) $\ell_{0}$ [20]; (**g**) localBF [12]; and (**h**) globalBF [12], respectively. The second row shows the zoomed-in view.

In Figure 6, we demonstrate the comparison results for a non-CAD mesh with rich details. As we can see in Figure 6d,g,h, the three filtering methods (RHM, localBF, and globalBF) blur fine details more or less. In contrast, TV and $\ell_{0}$ sharpen fine details in some extent; see Figure 6e,f. This situation is more serious for $\ell_{0}$ for its highest sparsity requirement. Moreover, both our method TS and HO can produce visually satisfactory results; see Figure 6b,c. However, from quantitative comparisons which will be presented in Section 3.3, we can see that the metric errors of our method are always lower than those of HO. Thus, our method outperforms the other compared state-of-the-art methods for non-CAD meshes with rich details.

To further testify the effectiveness of our method, we perform it on a variety of real scanned meshes captured by the laser scanner and Kinect sensors. The comparison results for scanned data are presented in the following paragraphs. It should be mentioned that the scanned data in Figures 8–10 are provided by Wang et al. [26].

**Figure 7:**
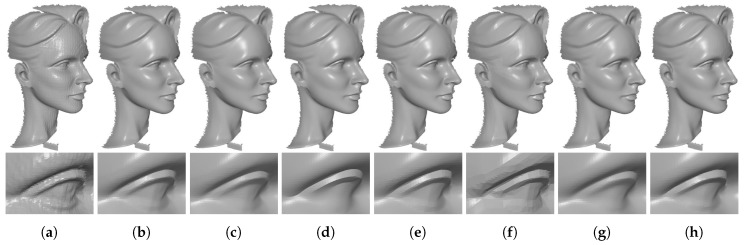
Denoising results of Wilhelm acquired by the laser scanner. From left to right: (**a**) noisy mesh, denoising results produced by (**b**) our method TS; (**c**) HO [1]; (**d**) RHM [14]; (**e**) TV [21]; (**f**) $\ell_{0}$ [20]; (**g**) localBF [12]; and (**h**) globalBF [12], respectively. The second row shows the zoomed-in view.

Figure 7 demonstrates the denoising results for a mesh acquired by the laser scanner. As we can see, our method TS, HO, TV, and globalBF can generate visually well results; see Figure 7b,c,e,h. $\ell_{0}$ suffers staircase effects in smooth regions shown in Figure 7f, while RHM and localBF blur geometric features demonstrated in Figure 7d,g.

**Figure 8:**
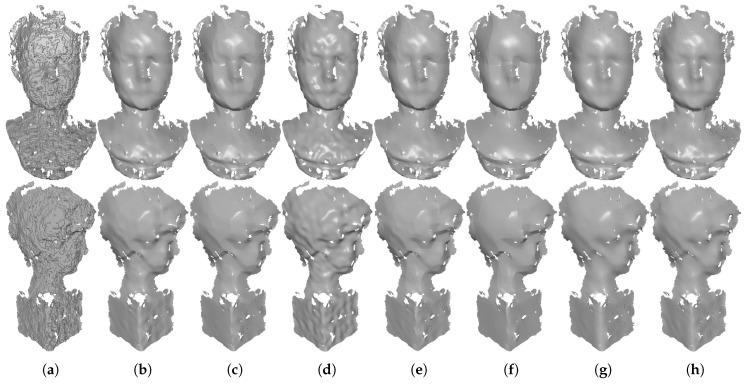
Denoising results of Boy and Girl captured by Kinect v1. From left to right: (**a**) noisy meshes, denoising results produced by (**b**) our method TS; (**c**) HO [1]; (**d**) RHM [14]; (**e**) TV [21]; (**f**) $\ell_{0}$ [20]; (**g**) localBF [12]; and (**h**) globalBF [12], respectively.

**Figure 9:**
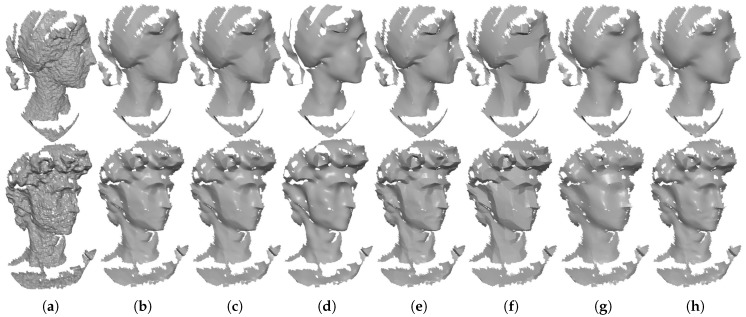
Denoising results of Big-Girl-01 and David captured by Kinect v2. From left to right: (**a**) noisy meshes, denoising results produced by (**b**) our method TS; (**c**) HO [1]; (**d**) RHM [14]; (**e**) TV [21]; (**f**) $\ell_{0}$ [20]; (**g**) localBF [12]; and (**h**) globalBF [12], respectively.

**Figure 10:**
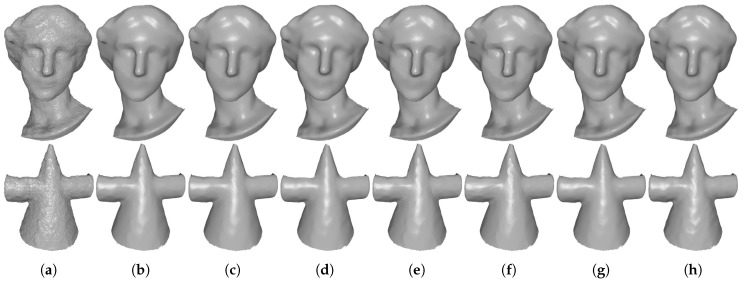
Denoising results of Big-Girl and Cone generated by Kinect-fusion process. From left to right: (**a**) noisy meshes, denoising results produced by (**b**) our method TS; (**c**) HO [1]; (**d**) RHM [14]; (**e**) TV [21]; (**f**) $\ell_{0}$ [20]; (**g**) localBF [12]; and (**h**) globalBF [12], respectively.

Figure 8 and Figure 9 demonstrate comparison results for single-frame meshes scanned by Kinect v1 and v2, respectively. As we can see in these two figures, except RHM which leaves some bumps in the denoising results, all of the tested methods can effectively remove the noise. Besides, TV and $\ell_{0}$ produce staircase effects in smooth regions; see the fifth and sixth columns of Figure 8 and Figure 9. This phenomenon is more severe for $\ell_{0}$. On the contrary, both localBF and globalBF blur some geometric features; see the seventh and eighth columns of Figure 8 and Figure 9. Apart from the above methods, both our method TS and HO can produce visually well results; see the second and third columns of Figure 8 and Figure 9. However, from the quantitative comparisons demonstrated in Section 3.3, we can find that metric errors of our method are always lower than those of HO. Figure 10 shows comparison results for the meshes generated by Kinect-fusion process. We can observe that our method TS, HO, RHM, localBF, and globalBF produce visually well results, whereas staircase effects still exist in the results produced by $\ell_{0}$ and TV. Again, from the quantitative comparisons shown in Section 3.3, we can see that metric errors of our method are always lower than those of the other compared methods.

### 3.3. Quantitative Comparisons

Besides the qualitative comparisons in the above subsection, the quantitative comparisons of our method and the state-of-the-art methods are given out in Table 1. The information of the tested meshes are listed in Table 2. Specifically, we use the mean square angular error (MSAE) and $L_{2}$ vertex-based error ($E_{v,2}$) to measure the fidelity of denoising results. These two error metrics are suggested in the works [1,12,21]. As we can see in Table 1, for all of the tested methods, the values of MSAE of our method are significantly smaller than all of the compared state-of-the-art methods. Moreover, in most cases, the results produced by our method have the least values of $E_{v,2}$. Thus, our method outperforms the other six typical methods quantitatively.

We also record CPU costs for all of the tested methods in Table 1. As we can see, RHM is the slowest method, whereas localBF is the fastest one. Our method TS is slower than globalBF, TV, and HO, but is faster than $\ell_{0}$. Although our method is a little computationally intensive, its computational time is still reasonable.

### 3.4. Comparisons of $\ell_{2}+\ell_{1}$ Fidelity with $\ell_{2}$ and $\ell_{1}$ Fidelities

In this subsection, we demonstrate effects of $\ell_{2}+\ell_{1}$ fidelity in the model (1) for removing impulsive and mixed noise. In order to show advantages of $\ell_{2}+\ell_{1}$ fidelity, we visually and quantitatively compare $\ell_{2}+\ell_{1}$ fidelity with $\ell_{2}$ and $\ell_{1}$ fidelities by the following configurations. We directly run the normal filtering model (1) for evaluating the performance of $\ell_{2}+\ell_{1}$ fidelity. We set β=0 in the model (1) to evaluate the performance of $\ell_{2}$ fidelity, while setting α=0 in the model (1) to testify the performance of $\ell_{1}$ fidelity.

**Figure 11:**
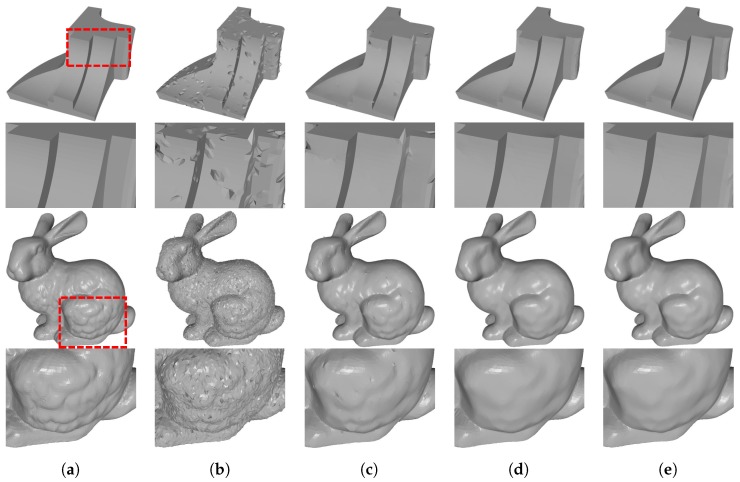
$\ell_{1}+\ell_{2}$ fidelity vs. $\ell_{2}$ and $\ell_{1}$ fidelities. From left to right: (**a**) clean meshes; (**b**) noisy meshes, denoising results produced by (**c**) $\ell_{2}$ fidelity; (**d**) $\ell_{1}$ fidelity; and (**e**) $\ell_{1}+\ell_{2}$ fidelity. Fandisk in the first row is corrupted with 10% of impulsive noise with scale of 0.7 mean edge length, while Bunny in the third row is corrupted with 10% of impulsive noise with 0.6 edge length and Gaussian noise with standard deviation σ=0.15 mean edge length. The even rows are zoomed-in views.

We show the denoising results produced by these three fidelities in Figure 11, and demonstrate the corresponding quantitative comparison results in Table 3. Based on these, we can reach the following conclusions. $\ell_{2}$ fidelity cannot do a good job in removing impulsive and mixed noise; see Figure 11c. In contrast, both $\ell_{2}+\ell_{1}$ and $\ell_{1}$ fidelities can efficiently remove large scale impulsive and mixed noise while preserving geometric features; see Figure 11e,d. However, the results produced by $\ell_{2}+\ell_{1}$ fidelity have lower numerical errors than those produced by $\ell_{1}$ fidelity. As a result, $\ell_{2}+\ell_{1}$ fidelity outperforms the other two fidelities.

## 4. Conclusions

In this paper, we propose a novel method to remove noise based on a triple sparsity prior. The problem is effectively solved by augmented Lagrangian method and variable-splitting. We discuss and compare our method with six state-of-the-art methods in various aspects. The experiments show that our method soundly outperforms the compared methods for both synthetic and scanned meshes at reasonable CPU costs.

## Figures and Tables

**Table 1:** Quantitative evaluation results of Figure 5, Figure 6, Figure 7, Figure 8, Figure 9 and Figure 10 for all of the test methods.

Mesh	$\mathrm{MSAE}(\times 10^{-3})$, $E_{v,2}\left(\times 10^{-3}\right)$; Costs (s)						
	TS	HO	RHM	TV	$\ell_{0}$	localBF	globalBF
Block	3.84, 1.63; 3.01	5.25, 2.00; 3.2	4.20, 3.20; 14.0	5.10, 1.82; 1.02	5.70, 2.47; 8.19	12.5, 3.22; 0.31	9.80, 2.74; 1.48
Joint	2.21, 1.34; 4.32	3.24, 1.78; 10.2	4.02, 2.38; 39.6	3.20, 2.24; 1.94	3.99, 1.93; 26.4	6.40, 2.52; 0.91	6.30, 3.55; 8.44
Max-Planck	18.5, 1.25; 66.3	26.8, 1.42; 23.2	25.6, 2.65; 99.0	29.0, 1.35; 11.9	33.0, 1.90; 56.6	25.8, 2.40; 1.98	20.2, 2.02; 3.94
Boy	57.9, 5.59; 10.4	62.3, 5.70; 7.39	65.13, 7.07; 33.6	71.1, 5.29; 5.69	63.6, 5.75; 13.9	61.6, 5.86; 0.48	73.1, 6.09; 0.85
Girl	80.4, 10.6; 22.6	82.5, 10.8; 13.1	85.0, 11.0; 56.2	85.1, 11.1; 11.0	84.4, 10.2;41.9	80.9, 10.9; 1.11	81.3, 10.6; 2.53
Big-Girl-01	66.0, 10.3; 2.01	70.5, 11.2; 1.98	75.5, 12.5; 7.89	66.4, 10.6; 3.31	82.2, 10.6; 3.87	66.3, 11.8; 0.13	68.9, 10.5; 0.23
David	129, 8.45; 2.03	139, 8.85; 1.57	163, 11.2; 7.22	151, 8.80; 1.38	160, 9.19; 2.94	143, 8.80; 0.11	136, 9.54; 0.18
Big-Girl	36.4, 5.39; 16.0	39.1, 5.48; 12.5	39.8, 5.60; 58.8	38.7, 5.65; 6.68	39.6, 5.60; 19.9	36.6, 5.48; 1.08	36.5, 5.47; 2.38
Cone	53.1, 9.03; 12.2	53.5, 9.18; 7.29	63.0, 8.41; 33.7	55.4, 8.71; 6.42	54.6, 9.28; 8.73	56.7, 9.36; 0.68	58.2, 9.30; 1.15

**Table 2:** The numbers of vertices and faces of the meshes tested in Section 3.3.

Mesh	Block	Joint	Max-Planck	Boy	Girl	Big-Girl-01	David	Big-Girl	Cone
Vertices	8771	20,902	50,002	28,187	21,323	8698	7362	46,132	31,159
Faces	17,550	41,808	99,999	53,229	40,751	16,025	13,445	91,112	61,301

**Table 3:** Quantitative evaluation results of Figure 11.

Mesh	$\mathrm{MSAE}\left(\times 10^{-3}\right),\;E_{v,2}\left(\times 10^{-3}\right)$					
	$\ell_{2}$ Fidelity		$\ell_{1}$ Fidelity		$\ell_{1}+\ell_{2}$ Fidelity	
Fandisk	31.3,	8.03	5.50,	0.93	4.24,	**0.89**
Bunny	33.4,	**1.60**	23.0,	2.47	22.3,	2.41

## Abbreviations

The following abbreviations are used in this manuscript:

$E_{f_{2}}$	$\ell_{2}$-norm fidelity term
$E_{f_{1}}$	$\ell_{1}$-norm fidelity term
$E_{tv}$	TV (total variation) regularization term
$E_{aho}$	Anisotropic high order regularization term
a(·)	The area of (·)
len(·)	The length of (·)
∇	Gradient operator
D	Anisotropic second order operator
